# Poplar aquaporin *PIP1;1* promotes *Arabidopsis* growth and development

**DOI:** 10.1186/s12870-021-03017-2

**Published:** 2021-06-03

**Authors:** Huani Leng, Cheng Jiang, Xueqin Song, Mengzhu Lu, Xianchong Wan

**Affiliations:** 1 Institute of New Forestry Technology, Chinese Academy of Forestry, Beijing, 100091 China; 2 State Key Laboratory of Tree Genetics and Breeding, Key Laboratory of Tree Breeding and Cultivation of the National Forestry and Grassland Administration, Research Institute of Forestry, Chinese Academy of Forestry, Beijing, 100091 China; 3 Zhejiang Agriculture & Forestry University, Hangzhou, 311300 China; 4 Co-Innovation Center for Sustainable Forestry in Southern China, Nanjing Forestry University, Jiangsu, 210037 China

**Keywords:** *PtoPIP1;1*, Flowering time, Autonomous pathway, Growth, Turgor pressure

## Abstract

**Background:**

Root hydraulic conductance is primarily determined by the conductance of living tissues to radial water flow. Plasma membrane intrinsic proteins (PIPs) in root cortical cells are important for plants to take up water and are believed to be directly involved in cell growth.

**Results:**

In this study, we found that constitutive overexpression of the poplar root-specific gene *PtoPIP1;1* in *Arabidopsis* accelerated bolting and flowering. At the early stage of the developmental process, *PtoPIP1;1* OE *Arabidopsis* exhibited faster cell growth in both leaves and roots. The turgor pressure of plants was correspondingly increased in *PtoPIP1;1* OE *Arabidopsis,* and the water status was changed. At the same time, the expression levels of flowering-related genes (*CRY1*, *CRY2* and *FCA*) and hub genes in the regulatory networks underlying floral timing (*FT* and *SOC1*) were significantly upregulated in OE plants, while the floral repressor *FLC* gene was significantly downregulated.

**Conclusions:**

Taken together, the results of our study indicate that constitutive overexpression of *PtoPIP1;1* in *Arabidopsis* accelerates bolting and flowering through faster cell growth in both the leaf and root at an early stage of the developmental process. The autonomous pathway of flowering regulation may be executed by monitoring developmental age. The increase in turgor and changes in water status with *PtoPIP1;1* overexpression play a role in promoting cell growth.

**Supplementary Information:**

The online version contains supplementary material available at 10.1186/s12870-021-03017-2.

## Background

Plant growth derives from the meristem, and meristem cells divide for several cycles before expansion to their final volume [1]. The rate of individual cell expansion is considered to be a function of turgor pressure, cell wall properties and cell hydraulic conductivity [2, 3]. Sustained cell growth requires sufficient water; thus, cellular water movement and homeostasis are tightly controlled. Cellular water movement is mainly mediated by aquaporins (AQPs), which facilitate passive exchange of water across membranes [4]. The plasma membrane (PM) intrinsic protein (PIP) subfamily is a member of the AQP family [5]. Since the hydrophobic Casparian strip of the endodermal cells in the root severely restricts the radial transport of water, PIPs are regarded as the first threshold of radial water absorption on PM, responsible for up to 30%—90% of the root water permeability in more than ten plant species [5–7].

Given the indispensability of water for plant growth and the importance of PIP in water permeability, PIP should have a role in plant root growth. Delayed lateral root emergence was found in *Arabidopsis pip2;1* [8], as an example. Given the large members in the PIP family and their functional redundancy, some *PIP* mutations show no significant differences in growth morphology, such as *Arabidopsis pip1;2–1*, *pip1;2–2*, *pip2;2–1* and *pip2;2–2* [9, 10], which makes it hard to investigate their role in plant growth. Lower root total length, surface area, and root volume and fewer root tips were found in *OsPIP2; 1* RNAi transgenic plants [11], and obvious morphological deformation and developmental retardation were observed in *BnPIP1* antisense transgenic tobacco [12], which provides evidence that PIP is involved in plant growth and development. Recently, much work has been done to improve plant growth via the genetic manipulation of PIPs. Although not all attempts were successful, better growth performance was observed in some PIP-overexpressing (OE) plants. Listed below are a few examples: *VvPIP2;4 N* OE grape [13], *TdPIP2;1* OE wheat [14], *RsPIP2;1* OE *Eucalyptus* [15], *AtPIP1b*, *TdPIP1;1* and *TdPIP2;1* OE tobacco [16, 17], *RcPIP2;1*, *RcPIP2;2*, and *AcPIP2* OE *Arabidopsis* [18, 19].

Turgor pressure is an essential driver of plant cell growth. Sustained cell growth requires sufficient water to keep the stable turgor pressure above the yield threshold, while turgor pressure provides the physical driving force against the cell wall and thus promotes cell division [20, 21] and cell expansion [22–24]. Early in 1965, the relationship between the steady-state elongation rate and turgor pressure was recognized [25]. Later, it was reported that cell division was greatly stimulated as turgor pressure increased to 1.0 bar, while cell expansion was stimulated as turgor pressure increased above 3.0 bar in isolated radish (*Raphanus sativus* L., var. Red Prince) cotyledons [20].

**Fig. 1:**
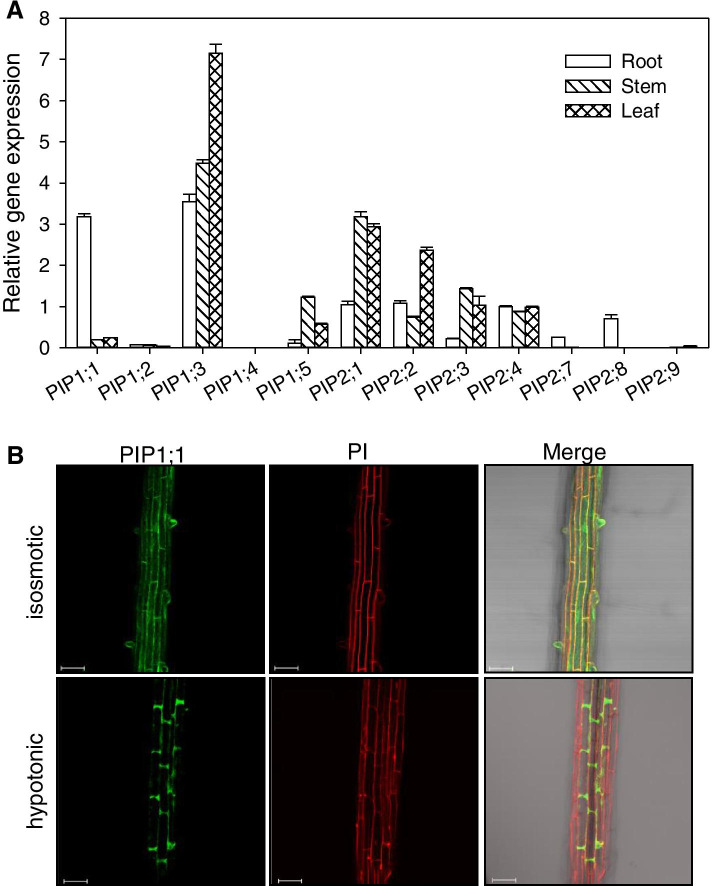
*PIP* gene expression in poplar tissues and subcellular localization of PtoPIP1;1. **a** The relative expression level of *PIP* genes in different tissues. *EIF-5A* was used as the internal control. Bars represent the standard error of the mean of three biological replicates. Means ± standard errors (*n* = 3) are shown. **b** Subcellular localization of PtoPIP1;1 in PIP1;1-YFP transgenic *Arabidopsis* under isosmotic conditions and hypotonic conditions. Bars = 50 µm

In an early study, we found that root pressure participated in refilling the embolized vessels of poplar [26]. In this study, we conducted an experiment to detect the expression of poplar PIP genes in roots, stems and leaves through qRT-PCR, and the results showed that PIP1;1 and PIP2;8 were relatively highly expressed in roots compared to stems and leaves, and the relative expression level of PIP1;1 in roots was the highest (Fig. 1a). It should be noted that in this experiment, *PtPIP2,5* and *PtPIP2;6* were not included because the sequence similarity was very high, 99%, and no suitable primers were found when we conducted this experiment. However, later, a colleague in our lab found primers to successfully separate the two PIPs and determined that *PtPIP2;5* and *PtPIP2;6* are the most highly expressed PIPs in poplar roots [27]. qRT-PCR in this paper was carried out using two-month-old cutting poplar plants growing in a greenhouse, while Jiang et al. used tissue culture plantlets [27]. Despite the different growth conditions of the plants, the qRT-PCR results in this paper are consistent with those of the previous paper. Originally, we intended to investigate the involvement of the predominantly expressed aquaporins in root pressure. Thus, we conducted constitutive overexpression (OE) of the *Populus* PIP aquaporin *PtoPIP1;1* with *PtoPIP2;8* in *Arabidopsis* and unexpectedly found that *PtoPIP1;1* OE resulted in early flowering. To our knowledge, it has not been reported that AQP accelerates flowering. In addition to *PtoPIP1;1*, we also chose *PtoPIP1;3* and *PtoPIP2;3* for comparison. Our choice depends on different amino acid residues in loopE of PtoPIP1;1, PtoPIP1;3 and PtoPIP2;3. The different amino acid residues in loopE are related to their different interactions with PIP2, resulting in different water permeability coefficients of PIP2 [28]. Accordingly, the different protein sequences in loop E of PtoPIP1;1, PtoPIP1;3 and PtoPIP2;3 (Figure S6) may lead to different interactions with other PIPs and hence different water permeability coefficients.

Flowering is a typical event that occurs during the plant transition from vegetative growth to reproductive development, and five genetically defined pathways have been identified as controlling flowering: the vernalization pathway (VRN1, VRN3, etc.), the photoperiod pathway (CRY1, CRY2, etc.), the gibberellin pathway (GAI, RGA, SPY, etc.), the autonomous pathway (FCA, FPA, FVE, etc.) and the aging pathway [29]. LFY, FT, SOC1 and CO, however, are integrators in the regulatory networks underlying floral timing [29]. In this study, overexpression and wild-type plants grew in the same environment, so light or temperature could not be the cause of variation in flowering time. The autonomous pathway may monitor developmental age; plants must pass through a juvenile phase and reach the adult vegetative phase before they can flower [30]. Therefore, we hypothesize that the growth changes caused by aquaporins promote flowering in terms of aquaporin involvement in plant growth. Thus, we studied the growth rate and osmotic potential of transgenic plants to explore the mechanism by which AQP accelerates flowering. In a previous study, the effects of aquaporins on root hydraulic conductivity, growth, and even turgor pressure were inversely proven by inhibiting the activity of aquaporins with mercury [22, 31, 32]. In this study, we wanted to further explore the involvement of aquaporins in water conductivity, turgor pressure and growth by overexpressing aquaporins, as well as determining their relationship with flowering time.

## Results

### *PtoPIP1;1*-overexpressing* Arabidopsis* showed accelerated flowering

Fifteen *PIP* genes were identified in the genome of *P. trichocarpa* (Figure S1 and Table S1). The expression of poplar *PIP* genes in roots, stems and leaves was analyzed through qRT-PCR, and the results showed that *PIP1;1*, *PIP2;7* and *PIP2;8* were relatively highly expressed in roots among roots, stems and leaves, while the relative expression level of *PIP1;1* in roots was the highest (Fig. 1a). Then, *PIP1;1* was cloned from *P. tomentosa,* and its subcellular localization was investigated. Through transiently expressing the PtoPIP1;1-YFP fusion protein in leaf epidermal cells of *N. benthamiana*, the fluorescence signal of PtoPIP1;1-YFP merged well with FM4-64 and the endoplasmic reticulum (ER) marker HDEL, indicating that PtoPIP1;1 localized on the plasma membrane (PM) and ER (Figure S2). Consistent with the results, the localization of PtoPIP1;1 on PM was also observed in PtoPIP1;1-YFP transgenic *Arabidopsis* (Fig. 1b). Interestingly, under hypotonic conditions (water treatment), most PtoPIP1;1-YFP fluorescence signals were distributed at both ends of root epidermal and cortical cells (Fig. 1b), showing a polar-like localization pattern at the PM. This localization pattern of PtoPIP1;1 may reflect its dynamic response to different water conditions in facilitating water transport.

**Fig. 2:**
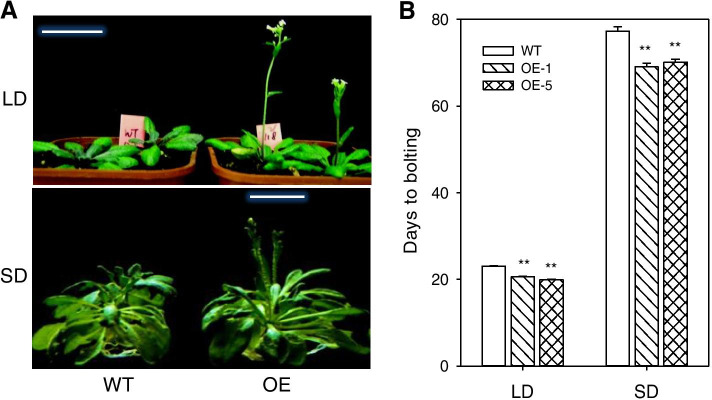
Early flowering of *PtoPIP1;1*-overexpressing (OE) *Arabidopsis*. **a** Photographs of wild-type (WT) and *PtoPIP1;1* OE *Arabidopsis* grown under long day (LD) and short day (SD) conditions. **b** Days of bolting of WT and two *PtoPIP1;1* OE *Arabidopsis* lines (OE-1 and OE-5) under LD and SD conditions. Means ± standard errors (*n* = 20 or more) are shown. Data were analyzed statistically using Student’s *t-*test. **P < 0.01

**Fig. 3:**
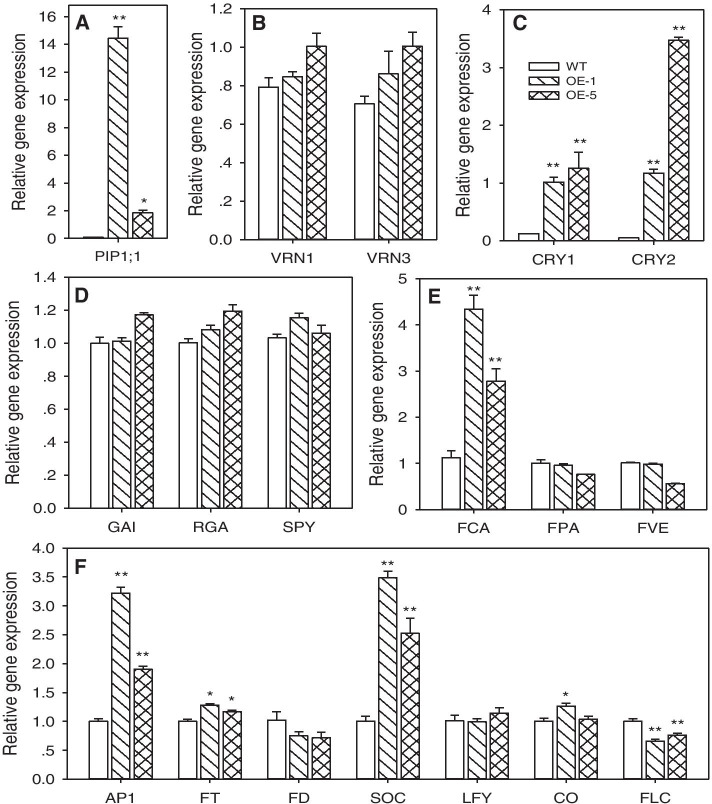
Expression of *PtoPIP1;1* and flowering-related genes in WT and two *PtoPIP1;1* OE *Arabidopsis* lines (OE-1 and OE-5). **a** The expression of *PtoPIP1;1*. **b-e** The expression levels of four flowering time pathway-related genes. **f** The expression levels of major hub genes in the regulatory networks underlying floral timing. The 14-DAS plants after 8 h of light (*n* = 3) were measured, and *Actin2* was used as the internal control. Data were analyzed statistically using Student’s *t-*test. *P < 0.05. **P < 0.01. PIP (plasma membrane intrinsic protein); VRN (vernalization response); CRY (cryptochrome); GAI (gibberellin-insensitive); RGA (repressor of GA1-3); SPY (spindly); FCA (an RNA-binding protein); FPA (an RNA-binding protein); FVE (a WD-40 repeat protein); AP1 (apetala1); FT (flowering locus t); FD (flowering locus d); SOC (suppressor of overexpression of constans 1); LFY (leafy); CO (constans); FLC (flowering locus c)

Compared to wild-type (WT), *PtoPIP1;1* OE *Arabidopsis* showed an early-flowering phenotype regardless of whether it was grown under long-day (LD) or short-day conditions (SD) (Fig. 2). In addition, the OE PtoPIP1;1 lines had the same leaf number before bolting compared with nontransgenic control plants; however, the leaf development of the former was faster than that of the latter (Figure S3). In the developmental process (at 14 DAS), the expression levels of *CRY1*, *CRY2* and *FCA* (genes in photoperiod and autonomous pathways) as well as *FT* and *SOC1* (major hubs in the regulatory networks underlying floral timing) were significantly upregulated in OE plants, while the floral repressor gene *FLC* was significantly downregulated in OE plants (Fig. 3). Thus, overexpression of *PtoPIP1;1* was able to accelerate *Arabidopsis* development.

### Leaf cell growth

**Fig. 4:**
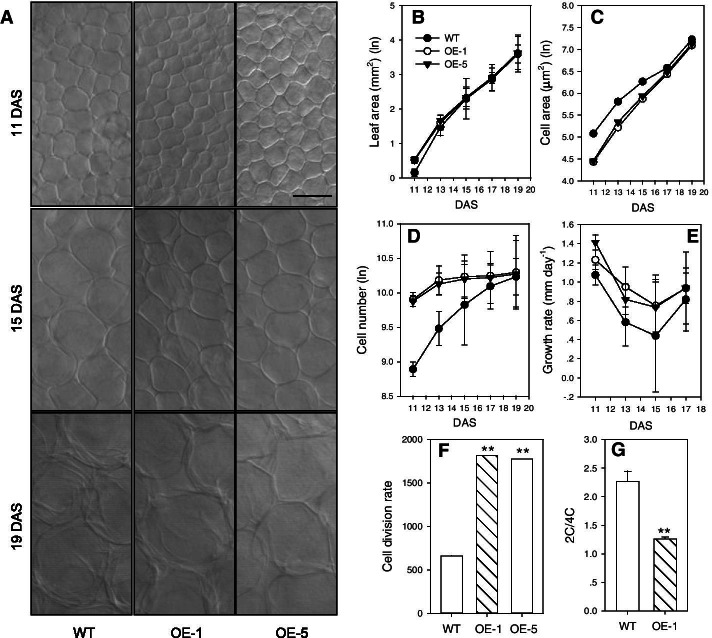
Characterization of the 3^rd^ leaf in WT and two *PtoPIP1;1* OE *Arabidopsis* lines (OE-1 and OE-5). **a** Palisade cells of the 3^rd^ leaf from WT and *PtoPIP1;1* OE at 11, 15, and 19 days after stratification (DAS). Bar = 20 µm. **b** Leaf area, Means ± standard errors (*n* = 6 ~ 8) are shown. **c** Palisade cell area. The cell area was measured using 40 to 60 leaf cells from photographs of 6 to 8 leaves. **d** The calculated cell number of the 3^rd^ leaf from WT and two *PtoPIP1;1* OE lines. Means ± standard errors (*n* = 6 ~ 8) are shown. **e** The relative cell growth rate was calculated based on the data from A. The relative cell growth rate is expressed as the increase in cell area (µm^2^) relative to the previous cell area per unit of time (day). **f** The average cell division rate was calculated based on the data from A. The average cell division rate of 11-DAS seedlings is expressed as the average cell number per unit of time. **g** The ratio of 2C/4C cells from the 3^rd^ leaf of WT and *PtoPIP1;1* OE lines at 11 DAS by flow cytometric analysis of their nuclear DNA content. Data for the ratio of 2C/4C represent the mean of six biological replicates with multiple leaves pooled in each replicate. Data were analyzed statistically using Student’s *t-*test. **P < 0.01

To determine the mechanism of accelerated cell growth, the number and size of cells during leaf development were kinematically analyzed. The 3^rd^ leaf was harvested every 2 days during the development process of WT and *PtoPIP1;1* OE *Arabidopsis*; more precisely, the sampling time was from 11 to 19 days after stratification (DAS). The leaf size and palisade cell area were measured. The leaf area of *PtoPIP1;1* OE plants was 45.3% and 17.5% larger than that of WT at 11 and 13 DAS, respectively, but comparable at 19 DAS (Fig. 4b). The palisade cell size of OE plants, however, was smaller than that of WT at 11, 13, and 15 DAS but similar at 17 and 19 DAS (Fig. 4a, c). The calculated cell number in OE plants was greater than that in WT at 11 and 13 DAS but nearly the same at 17 and 19 DAS (Fig. 4d). The higher cell division rate and larger leaf area of *PtoPIP1;1* OE *Arabidopsis* than of WT at the early developmental stage (Fig. 4e, f) indicate that *PtoPIP1;1* can promote leaf growth and development by accelerating cell division. Furthermore, the status of cell division of WT and *PtoPIP1;1* OE *Arabidopsis* was analyzed at 11 DAS by flow cytometry. The results showed that the 4C fraction in *PtoPIP1;1* OE *Arabidopsis* (55.6%) was much greater than that in WT Arabidopsis (31.5%) (Fig. 4g), which further indicates that PtoPIP1;1 could promote leaf cell growth.

### Root cell growth

**Fig. 5:**
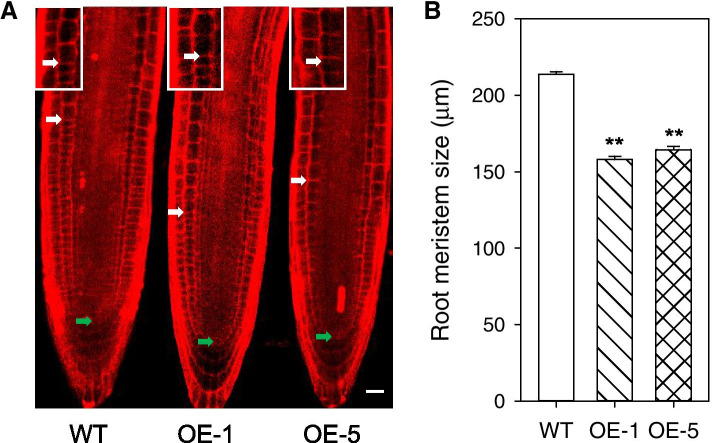
Root meristem zone in WT and two *PtoPIP1;1* OE *Arabidopsis* lines (OE-1 and OE-5). **a** Root meristem zone. Green and white arrowheads indicate the QC and the cortex TZ, respectively. The insert shows an enlarged view of elongating cells exiting from the meristem at the TZ. Bar = 10 µm. **b** Measurements of root meristem size represented in A. The root meristem size was measured with at least 20 roots in three independent experiments. Data were analyzed statistically using Student’s *t-*test. **P < 0.01

*Arabidopsis* roots are also a good model to study cell division, elongation and terminal differentiation [33]; thus, root length, root meristem size, and root cell size were investigated in both WT and *PtoPIP1;1* OE *Arabidopsis*. The root length of *PtoPIP1;1* OE *Arabidopsis* was not significantly different from that of WT (Figure S4A, B); however, the root meristem size of *PtoPIP1;1* OE *Arabidopsis* was significantly decreased by 24.5% (Fig. 5), indicating that root cell elongation-differentiation was promoted in *PtoPIP1;1* OE *Arabidopsis*. In addition, the cell length of the 6^th^ cell from elongating roots in *PtoPIP1;1* OE *Arabidopsis* was 28.9% smaller than that of WT, whereas the length of elongated cells was not significantly different between WT and *PtoPIP1;1* OE *Arabidopsis* (Figure S4C), which is consistent with the growth process of leaf palisade cells. The root length and root meristem size of another 2 *PIP*s, *PtoPIP1;3* and *PtoPIP2;3*, OE *Arabidopsis* showed no difference from that of WT (Figure S5), indicating that not all of the *PIP*s were involved in the regulation of root cell growth.

### Water loss and turgor pressure

**Fig. 6:**
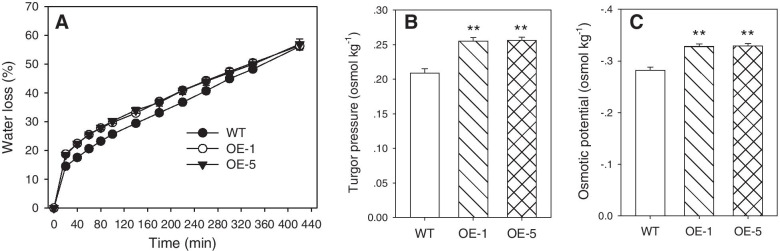
Physiological parameters detected in WT and two *PtoPIP1;1* OE *Arabidopsis* lines (OE-1 and OE-5). **a** Water loss detected in WT and *PtoPIP1;1* OE lines. Bars represent the standard error of the mean of at least 4 plants. **b, c** Turgor pressure (**b)** and osmotic potential (**c)** of WT and *PtoPIP1;1* OE lines at 11 DAS. Data are shown as the means ± SE from six biological replicates. Data were analyzed statistically using Student’s *t-*test. **P < 0.01

Since water movement across the cell membrane is facilitated by AQPs [34], the difference in water loss between WT and *PtoPIP1;1* OE *Arabidopsis* was examined. The water loss in *PtoPIP1;1* OE *Arabidopsis* was significantly faster than that in WT (Fig. 6a), indicating that overexpression of *PtoPIP1;1* changed the water status. Since turgor pressure plays pivotal roles in cell division and expansion/elongation [19, 24, 35], the osmotic potential of *PtoPIP1;1* OE *Arabidopsis* was measured, and turgor pressure was calculated accordingly. Compared with WT Arabidopsis, *PtoPIP1;1* OE *Arabidopsis* exhibited significantly lower osmotic potential and higher turgor pressure (Fig. 6b, c). Notably, the turgor pressure was increased by a value of approximately 0.05 osmol kg^−1^ (Fig. 6b), which is enough to promote cell division according to a previous study of radish cotyledons [19].

## Discussion

In this study, we report that constitutive overexpression of *PtoPIP1;1* in *Arabidopsis* accelerates plant development with rapid leaf and root cell growth. Turgor pressure was altered in *PtoPIP1;1* OE *Arabidopsis* to a level that can affect cell growth.

The autonomous pathway for controlling flowering refers to endogenous regulators that are independent of the photoperiod, temperature and gibberellin pathways. All genes in the autonomous pathway act by repressing *FLC* expression [29], as shown in this study, in which *FLC* expression was significantly downregulated in OE plants. FCA (its gene was upregulated in this study) was shown to be associated with the downregulation of *FLC* [36]. On the other hand, the upregulation of *FT* and *SOC1* also confirmed the downregulation of *FLC* [37]. In addition, cryptochromes (CRY) act to promote photomorphogenic development and the transition from vegetative to floral development [38]. The growth rate and growth amount may input signals to the flowering regulation pathway. As reported, *Agalinis strictifolia* flowered earlier than slower growing individuals [39], and larger plants flowered earlier and produced more flowers than smaller plants [40]. In this study, the earlier flowering *Arabidopsis* grew faster in both the root and leaf during the developmental process.

Through phenotypic and histocytological analysis, we found that *PtoPIP1;1* OE *Arabidopsis* exhibited the same accelerated growth as the previously mentioned *PIP* OE plants [13, 14, 16, 18, 41, 42]. However, it should be noted that not all *PIP* OE plants showed improved growth. For example, *AtPIP1;4* and *AtPIP2;5* OE *Arabidopsis* and tobacco [43], *MusaPIP1;2* and *MusaPIP2;6* OE banana [44, 45], *BnPIP1* OE tobacco [12], *CsPIP1;1*, *CfPIP2;1*, *MfPIP2-7*, *ThPIP2;5*, and *MsPIP2;2* OE *Arabidopsis* [43, 46–48] were indistinguishable from their nontransgenic controls. In addition, the growth of some *PIP* OE plants was even inhibited, such as *BjPIP1* OE tobacco [49], *RsPIP1;1* OE *Eucalyptus* [15], and *AtPIP2;1* OE *Arabidopsis* [8]. In our study, although *PtoPIP1;1* OE *Arabidopsis* exhibited accelerated plant growth, the growth and development of *PtoPIP1;3* and *PtoPIP2;3* OE *Arabidopsis* were indistinguishable from those of WT. Therefore, not all PIP members can improve plant growth.

Cell growth occurs through cell division and cell expansion/elongation-differentiation [1]. In our study, the growth process of leaf palisade cells and root cells in *PtoPIP1;1* OE *Arabidopsis* was similar. Leaf size was larger and palisade cell size was smaller in *PtoPIP1;1* OE *Arabidopsis* than in WT at the early stage of leaf development, while leaf size and palisade cell size became similar to WT at the later stage of leaf development, indicating that cell division was promoted at the early stage of leaf development and that cell expansion was promoted at the late stage of leaf development by the overexpression of *PtoPIP1;1*. For roots, although root meristem size was decreased in *PtoPIP1;1* OE *Arabidopsis*, the whole root length and the mature root cell size were similar between WT and *PtoPIP1;1* OE lines, indicating that root cell elongation-differentiation was enhanced by the overexpression of *PtoPIP1;1*. In summary, the overexpression of *PtoPIP1;1* accelerated leaf and root cell growth and thus leaf area growth at the early stage of the developmental process.

Turgor pressure is an essential driver of plant cell growth, including cell division, cell expansion and cell wall extension [19, 24, 35, 50]. In this study, the turgor pressure of *PtoPIP1;1* OE *Arabidopsis* was approximately 1.3 bar higher than that of WT. It was reported that cell division could be greatly stimulated as turgor pressure increased to 1.0 bar, while cell expansion was stimulated as turgor pressure increased above 3.0 bar in isolated radish cotyledons [20]. Accordingly, the increased turgor pressure of *PtoPIP1;1* OE *Arabidopsis* is enough to promote cell division. Although the underlying mechanism of turgor change in terms of *PtoPIP1;1* expression is not clear, some plant aquaporins can transport small neutral solutes, such as glycerol, urea, formamide, acetamide, methylammonium, boric acid, silicic acid, lactic acid and CO_2_ (see review by Maurel et al. [5]), which can all affect the osmolality and turgor of cells. For example, aquaporin-mediated hydraulic conductivity reduction in maize roots by arid load and hydrogen peroxide and anoxia application was reported to have an impact on turgor pressure and leaf elongation rate [22], and *CpPIP2* was suggested as a potential gene to adjust the turgor pressure-driven elongation of developing fibers in transgenic cotton [51]. In fact, the osmotic potential was decreased and the turgor pressure was increased in *PtoPIP1;1* OE *Arabidopsis*, although the turgor pressure here was calculated but not directly determined. However, it should be noted that the turgor pressure estimation method used here was approved in comparison to the direct turgor pressure determination method using the cell-pressure probe, and the result showed good agreement between these two methods [52]. Interestingly, with the same material, PtPIP1;1 was found to participate in the osmotic stress response [27].

In this study, *PtoPIP1;1* OE *Arabidopsis* exhibited accelerated plant growth, while the growth of *PtoPIP1;3* OE *Arabidopsis* was indistinguishable from that of WT. The water loss experiment reflected changes in the water status and water hydraulics of *PtoPIP1;1*. Thus, overexpression of *PtoPIP1;1* could increase the rate of passive water transport into roots, since all plants were grown on the medium in the plate with 100% relative humidity; under this condition, there was a considerable water potential gradient outside and inside the root. In addition, it was reported that WT plants may always suffer a water deficit due to suboptimal symplastic water transport via aquaporins even under favorable growth conditions [16]. Here, the accelerated plant growth observed in *PtoPIP1;1* OE *Arabidopsis* further confirmed the increased water permeability coefficient caused by PtoPIP1;1. In addition, under hypotonic conditions, most PtoPIP1;1 proteins accumulated in both ends of root epidermal and cortical cells. This localization pattern of PtoPIP1;1 may reflect its dynamic response to different water conditions in facilitating proper water transport. This finding is consistent with a previous report showing that the hydraulic conductivity of a growing root cell should be seen more in the context of facilitating water flow through a tissue rather than facilitating water uptake at a sufficiently high rate into an individual cell to support that cell’s expansion growth [53].

## Conclusions

Constitutive overexpression of the poplar root-specific gene *PtoPIP1;1* in *Arabidopsis* accelerates flowering through faster cell growth in both the leaf and root at an early stage of the developmental process. The results of this study indicate that expressed *PtoPIP1;1* can increase turgor pressure and that turgor pressure alteration promotes accelerated plant growth, at least at some stage of plant development. Accompanying accelerated growth, the genes involved in flowering regulation in the autonomous pathway changed. The autonomous pathway of flowering regulation may be executed by monitoring developmental age, and the expression levels of flowering-related genes (*CRY1*, *CRY2* and *FCA*) and hub genes in the regulatory networks underlying floral timing (*FT* and *SOC1*) are significantly upregulated in OE plants. It is likely that the promotion of leaf growth and development is the first message, and the hub regulatory networks of genes in the autonomous pathway constitute the second message.

## Methods

### Plant materials and growth conditions

*Arabidopsis thaliana* ecotype *Col-0*, obtained from the Arabidopsis Biological Resource Center (ABRC) through TAIR (www.arabidopsis.org), was grown in a greenhouse at 21 °C and 16-h light/8-h dark with a relative humidity of 70% and a light intensity of 110 μmol m^−2^ s^−1^. Sterilized seeds were plated on 1/2 MS supplemented with 1% sucrose (Suc) and 0.8% agar. Seven-DAS (days after stratification) seedlings were transferred to soil and further grown for approximately two months to harvest seeds. For meristem size analysis and water potential measurement, transgenic seedlings were grown together with their corresponding WT plants on the same plate placed vertically.

The seedlings of a hybrid poplar crossing *Populus alba* and *P. glandulosa* (clone 84 K) were propagated by stem cuttings. Sections of stems 8 cm long with two or three lateral buds were rooted and grown for 2 months in plastic pots (30 cm deep × 26 cm diameter, one seedling per pot) filled with artificial soils in a half-controlled greenhouse at 20 °C—28 °C with relative humidity and photosynthetically active radiation fluctuating between 30 and 55%, 600 and 1200 μmol m-2 s-1, respectively. Pots were randomly rotated twice a week to minimize edge effects.

### Subcellular localization

To determine the subcellular localization of PtoPIP1;1, attB sites were added via PCR-mediated ligation to the coding regions of PtoPIP1;1 without a stop codon and inserted into pDONR 222 according to the manufacturer’s protocol (Invitrogen). The cDNA was then transferred via LR reaction (Invitrogen) into the destination vector pEarleyGate 101(C-YFP), resulting in the 35S::PtoPIP1;1-YFP construct. Transient expression of PtoPIP1;1-YFP and GFP-HDEL in *Nicotiana benthamiana* leaf lower epidermal cells was performed using *Agrobacterium* transformation according to a previous report [54].

### Plasmid construction and gene expression analysis

*Populus* PIP (Table S1) sequences were obtained from the Poplar Genome Database (http://www.phytozome.net/poplar.php, release 3.0) and NCBI database (http://www.ncbi.nlm.nih.gov/), while *Arabidopsis* PIP protein sequences were obtained from TAIR (http://www.arabidopsis.org/). Then, amino acid sequence alignment was performed using the CLUSTALX program (http://www.clustal.org/). A putative full-length expressed sequence (locus name Potri.010G191900) homologous to other *PIP1s* was obtained (Figure S1). The CDSs of *PIP1;1*, *PIP1;3* and *PIP2;3* were cloned from *Populus tomentosa* using the primers listed in Table S2. For overexpression, the purified PCR products were first inserted into the pMD-19 vector (TaKaRa) and confirmed by DNA sequencing. Then, XbaI and SalI were used to digest and ligate the sequences of *PtoPIP1;1*, *PtoPIP1;3* and *PtoPIP2;3* into the pBI121 binary vector using T4 ligase (NEB), resulting in the *35S::PtoPIP1;1*, *35S::PtoPIP1;3* and *35S::PtoPIP2;3* constructs, respectively.

qRT-PCR in this paper was carried out using two-month-old cutting poplar plants (*Populus alba* × *Populus glandulosa*, clone 84 K) growing in a greenhouse. The third leaves from the apex were sampled for total RNA extraction, the xylem part from the middle portion of the stem was used for stem RNA extraction, and fine roots were used for root RNA extraction. Quantitative real-time PCR (qRT-PCR) analysis was carried out with a 7500 Real-Time PCR System (Applied Biosystems, CA, USA) using a SYBR Premix Ex TaqTM Kit (TaKaRa, Tokyo, Japan). Primers of *Arabidopsis* flowering-related genes [29] and PIP genes found in *P. trichocarpa* [26] for qRT-PCR analysis were designed with a length of 20–24 nucleotides and a predicted melting temperature of 59–61 °C (Table S3). PCRs were carried out in 20 µL final volumes containing 0.2 µM primers, 2 µL of cDNA and 10 µL of SYBR Premix Ex Taq™ (2 ×). The PCR program was 1 cycle of 30 s at 95 °C, followed by 40 cycles of 95 °C for 5 s and 60 °C for 34 s, with a final dissociation stage of 15 s at 95 °C, followed by 60 °C for 1 min and 95 °C for 15 s to verify the specificity of the primer pairs. PCRs without templates were used as negative controls. The final threshold cycle (Ct) values were the means of twelve values from three biological replicates with four technical replicates in each biological replicate. The relative transcript abundance was calculated using 7500 Real-Time PCR analysis software with *EIF-5A* for poplar [26] and *Actin2* for *Arabidopsis* [55] as an internal control.

### Plant transformation

*35S::PtPIP1;1*, *35S::PtPIP1;3*, *35S::PtPIP2;3*, and 35S::PtPIP1;1-YFP constructs were introduced into the *Agrobacterium tumefaciens* GV3101 strain. Agrobacterium-mediated transformation was performed using the floral dipping method [56] in *Col-0 Arabidopsis thaliana*. Putative transgenic plants were screened on 1/2 MS plates supplemented with 30 mg L^−1^ kanamycin and then transferred to soil for propagation. Kanamycin-resistant plants of the T2 generation were subjected to expression analyses. Homozygous T3 transgenic plants, together with WT plants, were used for further studies. In this study, a total of more than 10 overexpressing lines of *PtPIP1;1* were generated, and all of them had an early flowering phenotype compared to nontransgenic control plants. Among them, two independent lines, OE-1 and OE-5, were selected for further functional analyses given the heavy workload of the experiment.

For fluorescence signal detection, roots from *35S::PtPIP1;1*-YFP *Arabidopsis* were observed either in 1/2 MS or in water (hypotonic conditions) using a confocal microscope (Zeiss LSM 510). Images (Fig. 1b) shown are representative of at least three independent experiments with at least 10 roots in each solution.

### Leaf growth analysis

Leaf growth was analyzed on the 3rd leaf harvested at different time points (11, 15 and 19 DAS). Leaves were fixed in formalin-acetic acid-alcohol (FAA) and then cleared in chloral hydrate solution (200 g of chloral hydrate, 20 g of glycerol, 50 ml of H_2_O). Leaves were then mounted in 100% lactic acid on microscopy slides. All observations were performed using a Zeiss LSM 510 AX70 with a differential interference contrast objective. The leaf size and cell area were measured using photographs of 6 to 8 leaves and 40 to 60 leaf cells with IMAGEJ software (http://rsb.info.nih.gov/ij/), and cell numbers were calculated accordingly. The cell area was measured in the region located 25–75% from the distance between the tip and the base of the blade, halfway between the midrib and the leaf margin. For the kinematic analysis, ln-transformed means of leaf area, cell area, and cell number were locally fitted to a quadratic function, of which the first derivative was taken as the relative growth and expansion, respectively [57, 58].

### Flow cytometric analysis

Flow cytometric analysis was carried out according to a method reported previously [59]. The 3^rd^ leaves were dissected from *Arabidopsis* seedlings at 11 DAS and chopped with a razorblade in 400 µL of nuclear extraction buffer. After the addition of 600 µL of PI (propidium iodide) (50 μg/mL) staining buffer, the supernatant was collected after filtering with a 30-µm filter. The distribution of nuclear DNA content was analyzed with a FACSCalibur flow cytometer (BD Biosciences, CA, USA). For each sample, 10,000 events were analyzed. The cell cycle was analyzed using ModFit LT 3.0 software. Data for the ratio of 2C/4C represent the mean of six biological replicates with multiple leaves pooled in each replicate. The nuclear 2C/4C ratio reflects the status of the cells within the cell cycle [60].

### Root length and root-meristem size analysis

Roots were stained in a 10 µM PI solution (Sigma-Aldrich) for 2 min. The root meristem was defined as the zone of cortex cells extending from the quiescent center (QC) to the first elongated cell of the transition zone (TZ) [61]. Then, root meristem size was measured with an LSM 510 confocal microscope (Zeiss). Root length was measured using IMAGE J software (http://rsb.info.nih.gov/ij). Data on root length and root meristem size represent the means from three independent experiments with at least 20 roots.

### Water loss speed assay

To quantify water loss, at least four 14-DAS seedlings were acclimated in the dark for 3 h prior to measurement. Then, each whole seedling was weighed immediately and weighed every 20 min using a microbalance (the relative humidity was between 40 and 50% and the temperature was approximately 22 °C). The proportion of water loss was calculated on the basis of the initial fresh weight of the samples.

### Osmotic potential measurement and turgor pressure calculation

The 11-DAS seedlings were frozen in liquid nitrogen and then centrifuged for 20 min at 1,500 g in microcentrifuge tubes. Further separation of the cellular fluid from plant debris was obtained by centrifugation at 9,600 g for 10 min, and osmotic potential was measured using 30 µL samples at freezing-point depression with an Osmomat 030 (Gonotec, Berlin, Germany). Since the WT and *PtoPIP1;1* OE plants all grew in plates with 100% relative humidity, the water potential of the plants was used as the value of the osmotic media. Based on the above osmotic potential and water potential, the turgor pressure was calculated and could be regarded as an estimated value of turgor pressure in plants [62]. *PtoPIP1;1* OE *Arabidopsis* plants were grown together with their corresponding WT on the same plate. Osmotic potential was measured by using six biological replicates with multiple leaves pooled in each replicate.

## Supplementary Information

**Additional file 1**

**Additional file 2**

## Data Availability

All data generated or analyzed during this study are included in this article and available from the corresponding author on reasonable request.
