# Supplementary material for: Poplar aquaporin PIP1;1 promotes Arabidopsis growth and development
Source: BMC Plant Biol. 2021 Jun 3;21:253. doi: 10.1186/s12870-021-03017-2 (PMC8173918; doi:10.1186/s12870-021-03017-2)
Supplement: Supplementary file 1 — Additional file 1 [file 12870_2021_3017_MOESM1_ESM.pptx]

## Slide 1
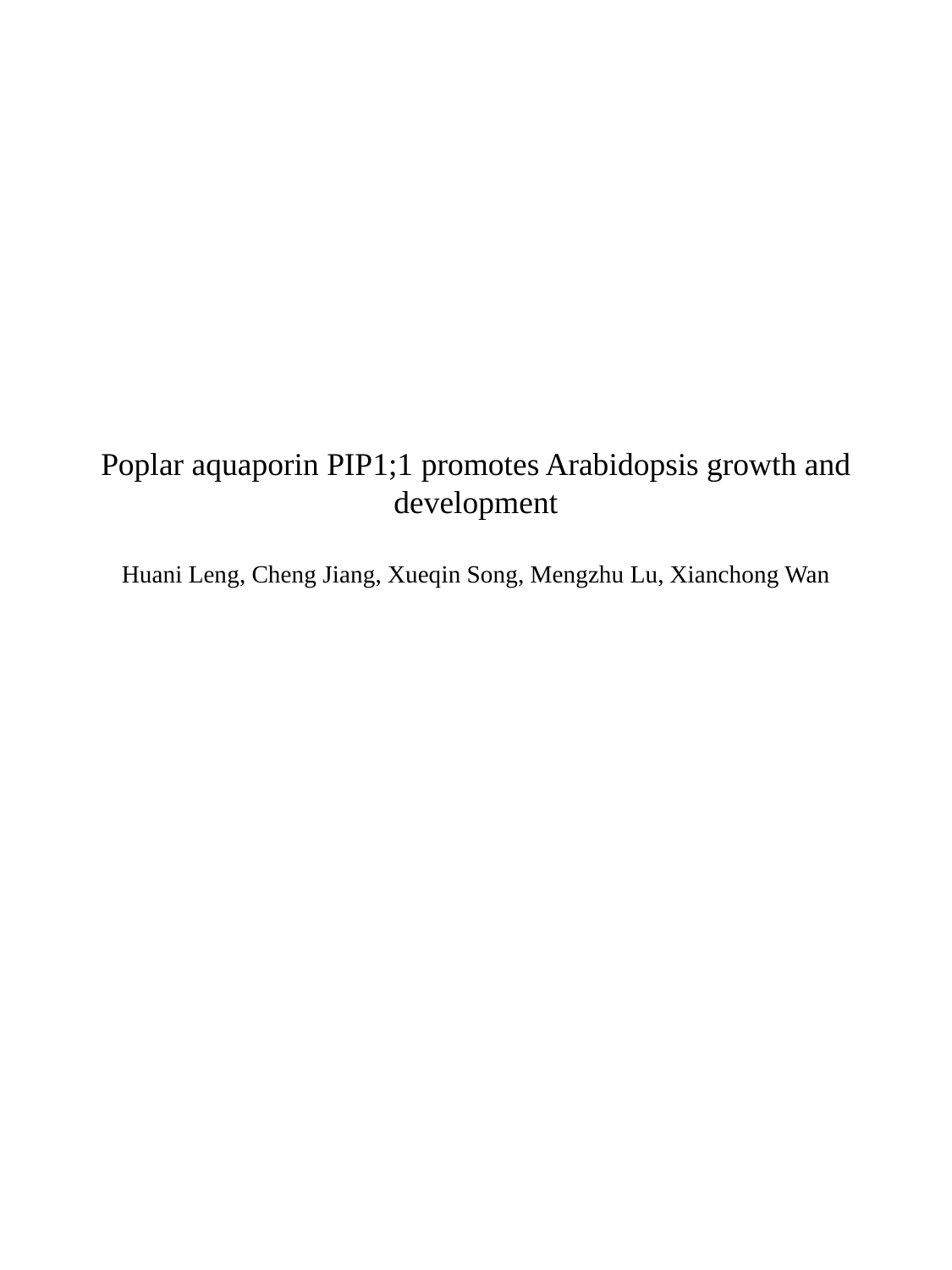

# Poplar aquaporin PIP1;1 promotes Arabidopsis growth and development Huani Leng, Cheng Jiang, Xueqin Song, Mengzhu Lu, Xianchong Wan

## Slide 2
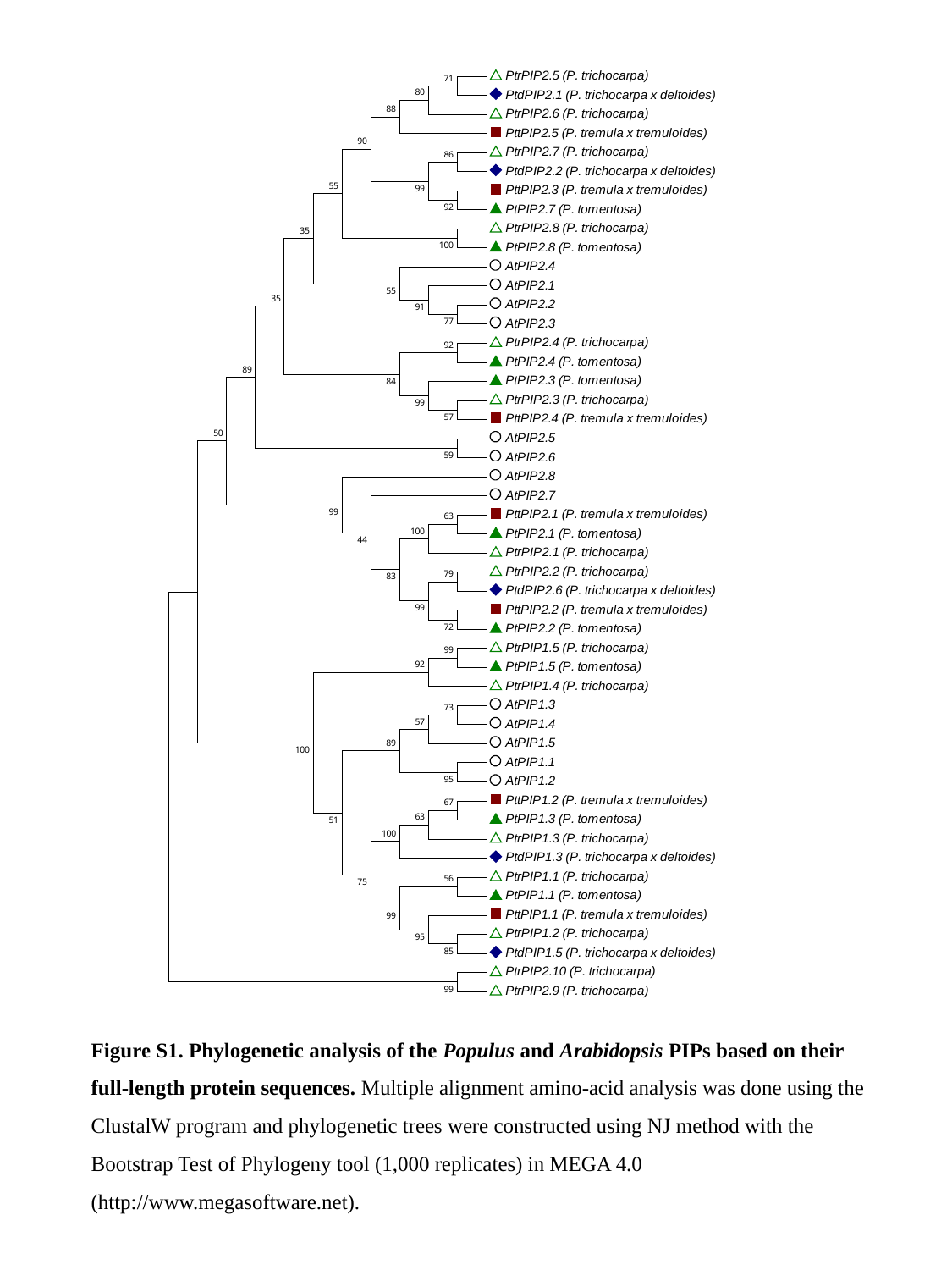

Figure S1. Phylogenetic analysis of the Populus and Arabidopsis PIPs based on their full-length protein sequences. Multiple alignment amino-acid analysis was done using the ClustalW program and phylogenetic trees were constructed using NJ method with the Bootstrap Test of Phylogeny tool (1,000 replicates) in MEGA 4.0 (http://www.megasoftware.net).

## Slide 3
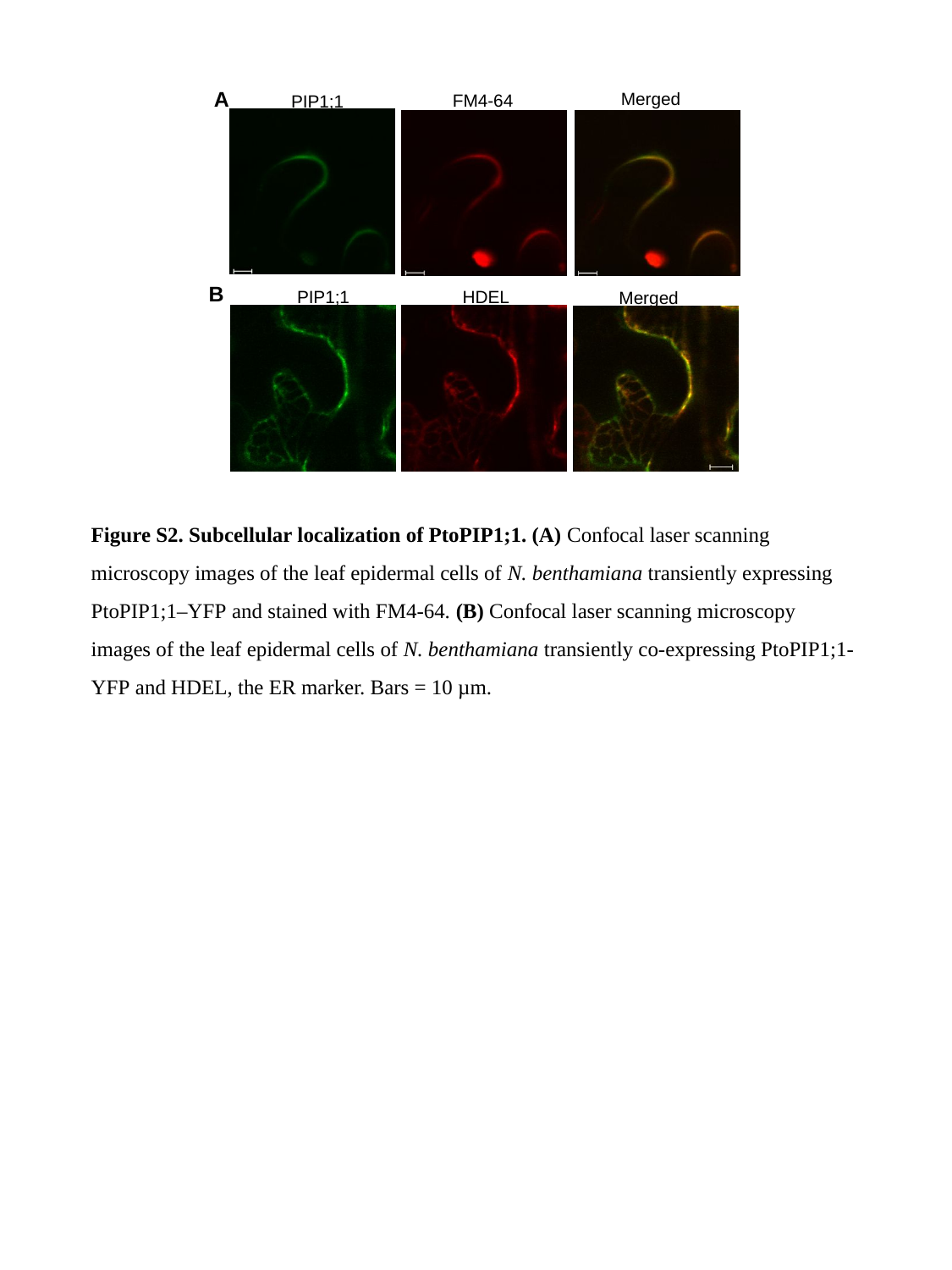

A
Merged
FM4-64
PIP1;1
B
PIP1;1
HDEL
Merged
Figure S2. Subcellular localization of PtoPIP1;1. (A) Confocal laser scanning microscopy images of the leaf epidermal cells of N. benthamiana transiently expressing PtoPIP1;1–YFP and stained with FM4-64. (B) Confocal laser scanning microscopy images of the leaf epidermal cells of N. benthamiana transiently co-expressing PtoPIP1;1-YFP and HDEL, the ER marker. Bars = 10 µm.

## Slide 4
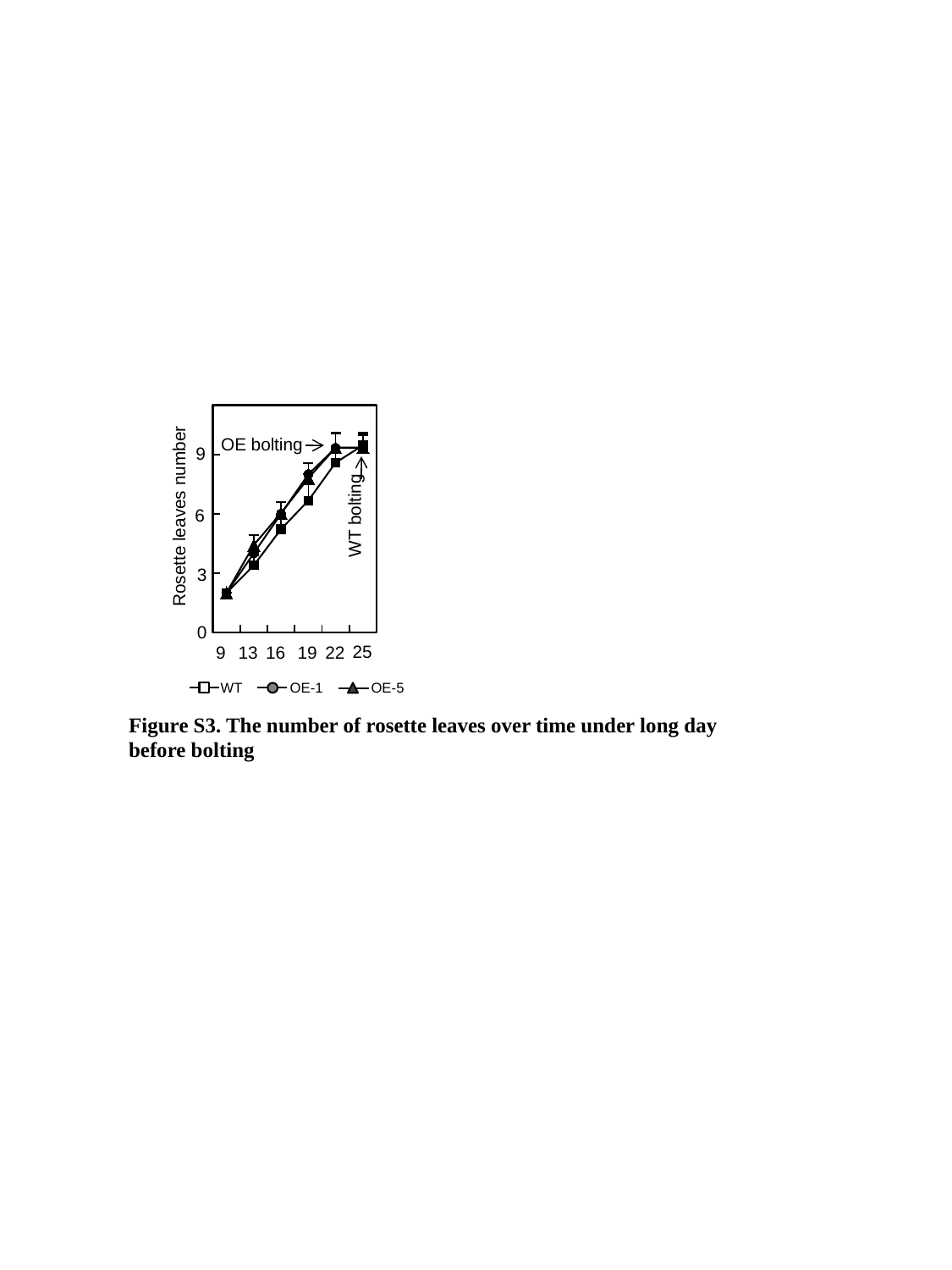

### Chart
| Category | WT | 35S:PtPIP1;1-1 | 35S:PtPIP1;1-8 |
|---|---|---|---|
| 9 | 2.0 | 2.0 | 2.0 |
| 13 | 3.3889 | 4.0 | 4.4167 |
| 16 | 5.2222 | 6.0 | 6.0417 |
| 19 | 6.6667 | 8.0 | 7.8 |
| 22 | 8.588235294117647 | 9.333333333333334 | 9.35 |
| 25 | 9.461538461538462 | 9.333333333333334 | 9.35 |OE bolting
9
WT bolting
6
Rosette leaves number
3
0
25
9
13
16
19
22
WT
OE-1
OE-5
Figure S3. The number of rosette leaves over time under long day before bolting

## Slide 5
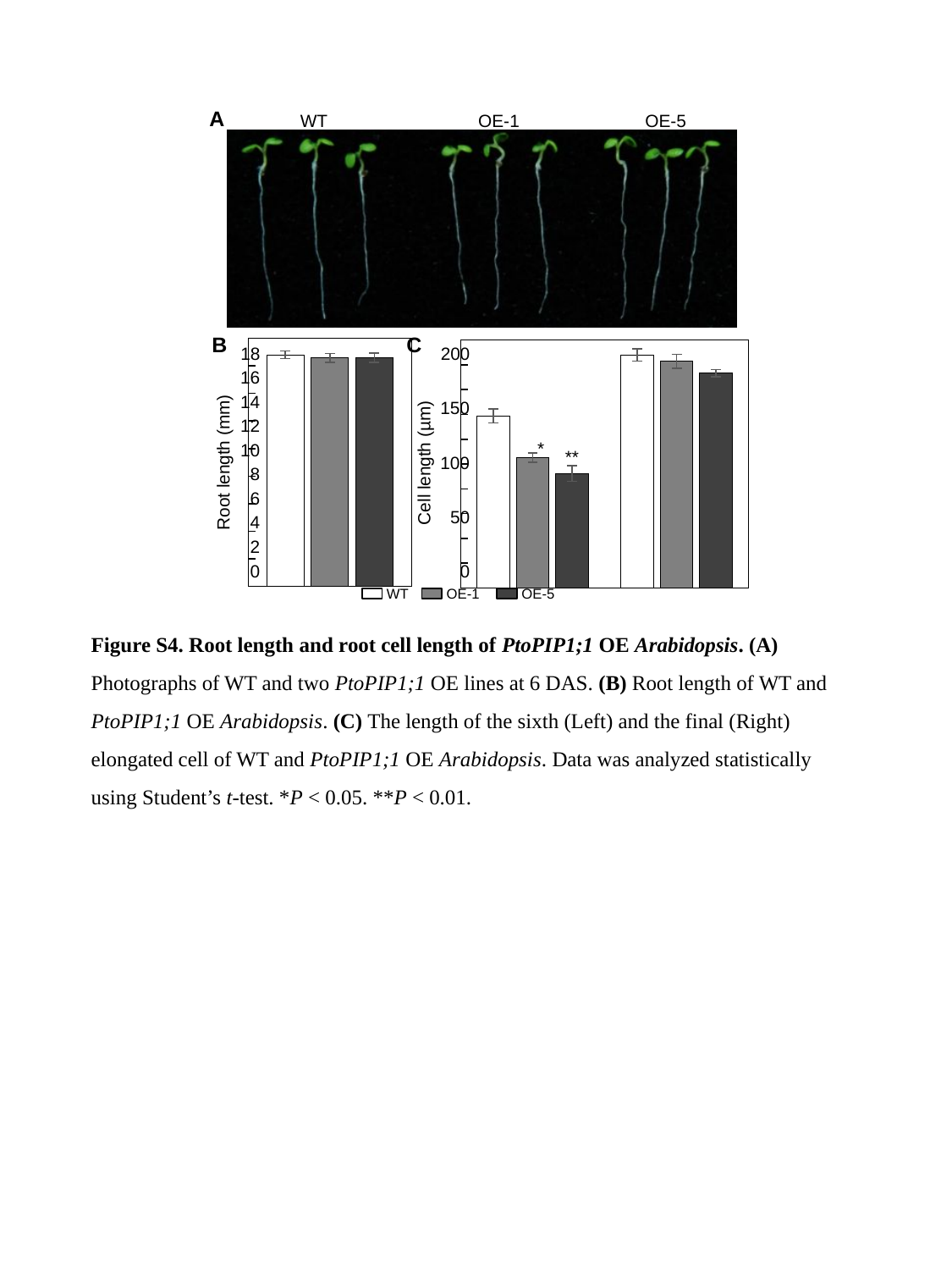

A
WT
OE-1
OE-5
B
C
### Chart
| Category | WT | OE-1 | OE-5 |
|---|---|---|---|
### Chart
| Category | WT | 35S:PtPIP1;1-1 | 35S:PtPIP1;1-5 |
|---|---|---|---|
| Tip | 138.7714 | 105.155 | 92.224 |
| Up | 187.9613 | 182.89 | 173.28 |18
200
16
14
150
12
*
10
**
100
Root length (mm)
Cell length (µm)
8
6
50
4
2
0
0
WT
OE-1
OE-5
Figure S4. Root length and root cell length of PtoPIP1;1 OE Arabidopsis. (A) Photographs of WT and two PtoPIP1;1 OE lines at 6 DAS. (B) Root length of WT and PtoPIP1;1 OE Arabidopsis. (C) The length of the sixth (Left) and the final (Right) elongated cell of WT and PtoPIP1;1 OE Arabidopsis. Data was analyzed statistically using Student’s t-test. *P < 0.05. **P < 0.01.

## Slide 6
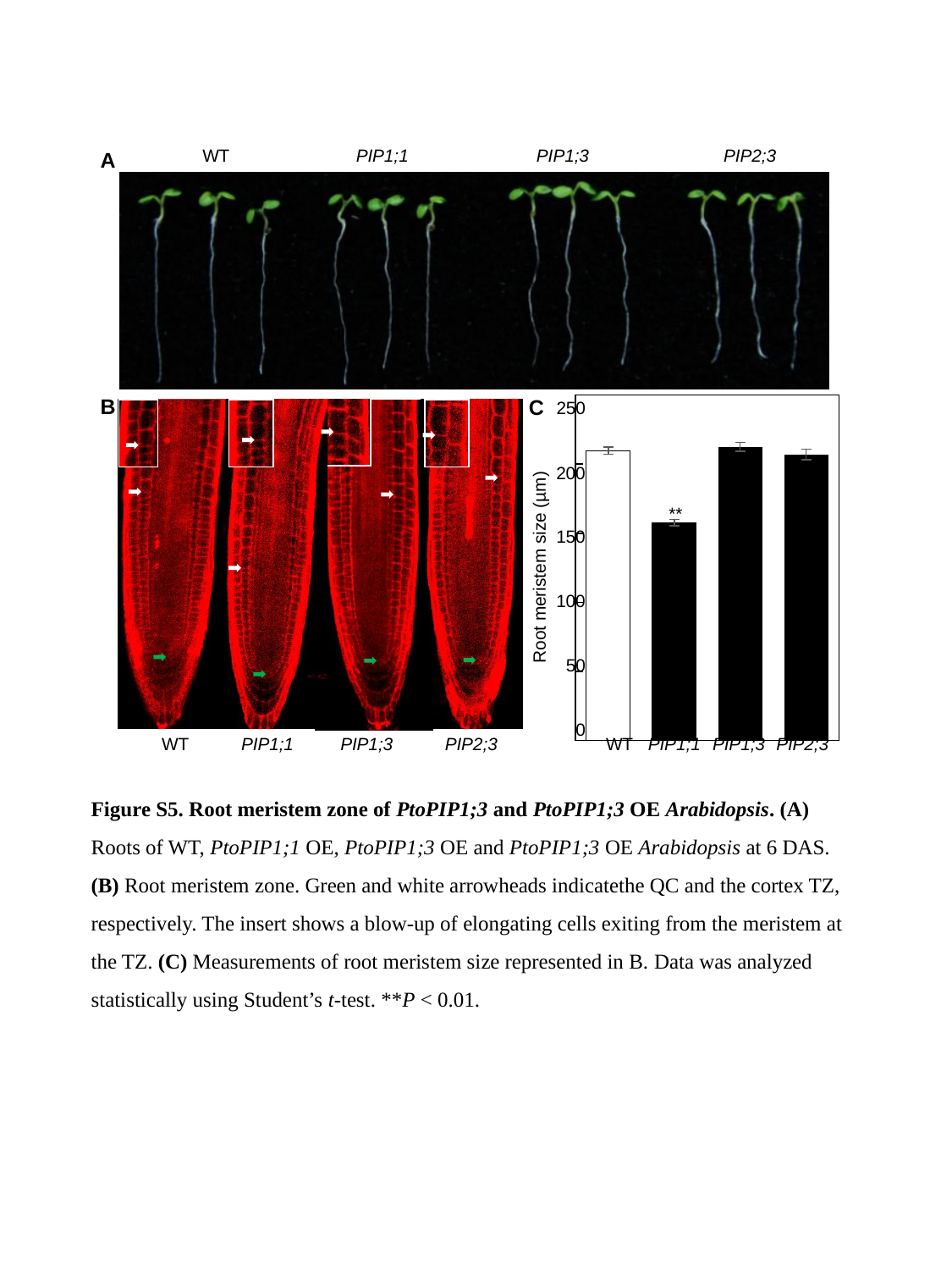

WT
PIP1;1
PIP1;3
PIP2;3
A
B
### Chart
| Category | |
|---|---|
| WT | 209.8016 |
| 1;1OE | 157.5958 |
| 1;3OE | 212.4232 |
| 2;3OE | 206.9889 |C
250
200
**
150
Root meristem size (µm)
100
50
0
WT
PIP1;1
PIP1;3
PIP2;3
WT
PIP1;3
PIP2;3
PIP1;1
Figure S5. Root meristem zone of PtoPIP1;3 and PtoPIP1;3 OE Arabidopsis. (A) Roots of WT, PtoPIP1;1 OE, PtoPIP1;3 OE and PtoPIP1;3 OE Arabidopsis at 6 DAS. (B) Root meristem zone. Green and white arrowheads indicatethe QC and the cortex TZ, respectively. The insert shows a blow-up of elongating cells exiting from the meristem at the TZ. (C) Measurements of root meristem size represented in B. Data was analyzed statistically using Student’s t-test. **P < 0.01.

## Slide 7
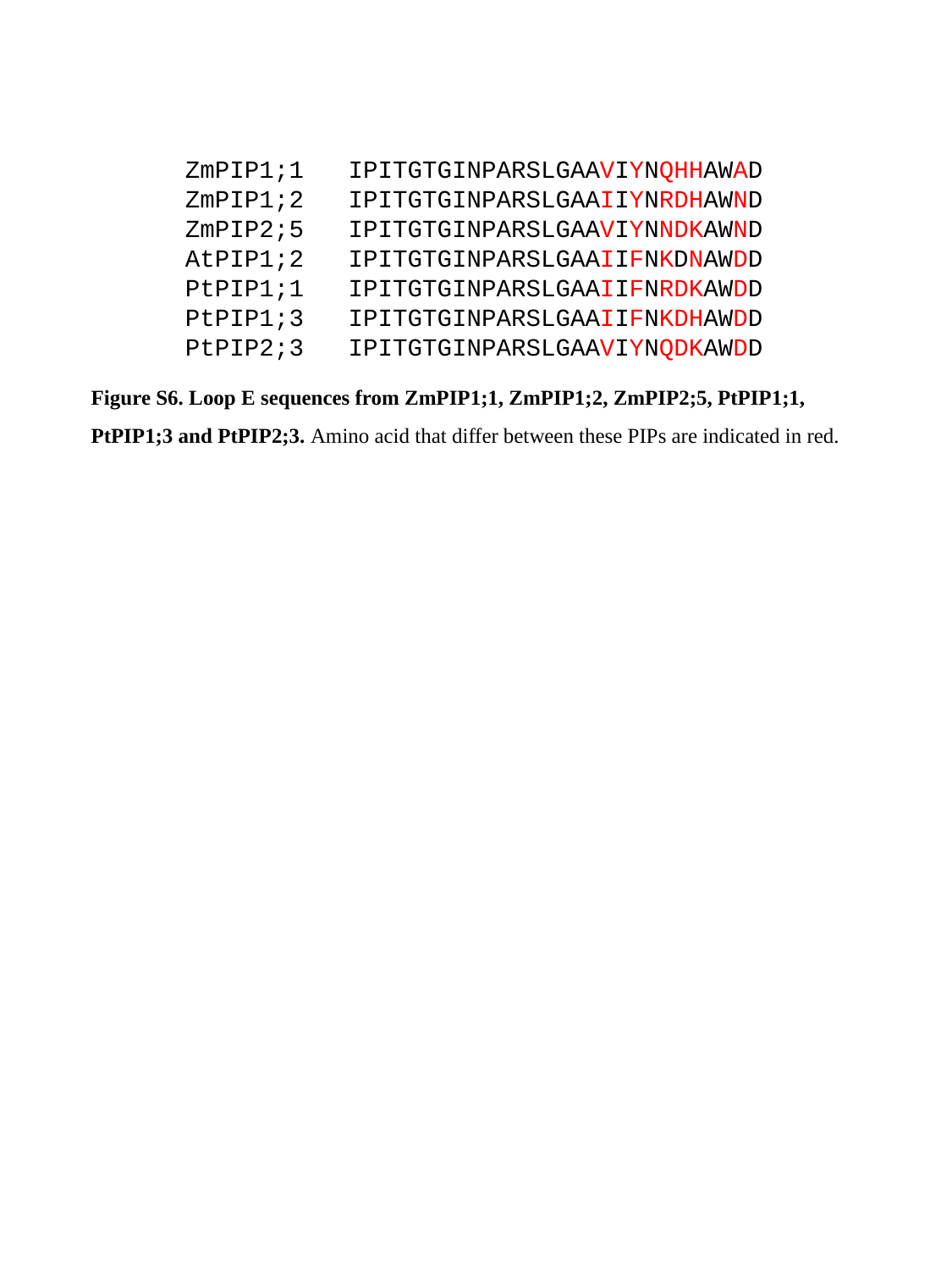

ZmPIP1;1 IPITGTGINPARSLGAAVIYNQHHAWAD
ZmPIP1;2 IPITGTGINPARSLGAAIIYNRDHAWND
ZmPIP2;5 IPITGTGINPARSLGAAVIYNNDKAWND
AtPIP1;2 IPITGTGINPARSLGAAIIFNKDNAWDD
PtPIP1;1 IPITGTGINPARSLGAAIIFNRDKAWDD
PtPIP1;3 IPITGTGINPARSLGAAIIFNKDHAWDD
PtPIP2;3 IPITGTGINPARSLGAAVIYNQDKAWDD
Figure S6. Loop E sequences from ZmPIP1;1, ZmPIP1;2, ZmPIP2;5, PtPIP1;1, PtPIP1;3 and PtPIP2;3. Amino acid that differ between these PIPs are indicated in red.
