# Supplementary material for: Poplar aquaporin PIP1;1 promotes Arabidopsis growth and development
Source: BMC Plant Biol. 2021 Jun 3;21:253. doi: 10.1186/s12870-021-03017-2 (PMC8173918; doi:10.1186/s12870-021-03017-2)
Supplement: Supplementary file 2 — Additional file 2 [file 12870_2021_3017_MOESM2_ESM.docx]

Poplar aquaporin *PIP1;1* promotes Arabidopsis growth and development

Huani Leng, Cheng Jiang, Xueqin Song, Mengzhu Lu, Xianchong Wan

Table S1. Basic information of PIP gene family in *Populus.*

| **Gene Name** | **Locus name** | **Gene length, ORF,introns** | **Chromosome location** |
| --- | --- | --- | --- |
| PtPIP1.1 | Potri.010G191900 | 1225,864,3 | Chr10: 18673115-18674816 |
| PtPIP1.2 | Potri.008G065600 | 1212,864,4 | Chr08: 3973880-3975728 |
| PtPIP1.3 | Potri.003G128600 | 2408,867,3 | Chr03: 14876395-14880577 |
| PtPIP1.4 | Potri.006G098100 | 1150,864,3 | Chr06: 7537631-7539399 |
| PtPIP1.5 | Potri.016G113300 | 1155,864,3 | Chr16: 11696553-11698374 |
| PtPIP2.1 | Potri.009G136600 | 1391,840,3 | Chr09: 10982790-10984508 |
| PtPIP2.2 | Potri.004G176300 | 1405,840,3 | Chr04: 19451568-19453491 |
| PtPIP2.3 | Potri.010G222700 | 2738,858,3 | Chr10: 20694148-20697432 |
| PtPIP2.4 | Potri.008G039600 | 3149,858,3 | Chr08: 2249258-2253276 |
| PtPIP2.5 | Potri.006G128000 | 1203,858,3 | Chr06: 10443014-10444501 |
| PtPIP2.7 | Potri.016G089500 | 1145,858,3 | Chr06:10449249-10450449 |
| PtPIP2.8 | Potri.009G013900 | 1486,855,3 | Chr16: 7248573-7250532 |
| PtPIP2.9 | Potri.005G109300 | 1273,765,3 | Chr09: 2254886-2256777 |
| PtPIP2.10 | Potri.005G109200 | 1330,870,3 | Chr05: 8392291-8395636 |

Table S2. Primers used for gene clone of PtoPIPs.

| Gene name | Forward primer | Reverse primer |
| --- | --- | --- |
| PIP1;1 | ATGGAGGGCAAAGAAGAAGAT | TCACAACCTGTGGTAAAGAGCT |
| PIP1;3 | ATGGAGGGCAAAGAAGAGGAT | TTAAGCTCTGCTCTTGAAAGGAAT |
| PIP2;3 | ATGGCAAAGGACATGGAAGTAG | TTAAAACCGTTGGGCGCTC |

Table S3. Primers used in qRT-PCR.

| Gene name | Forward primer（5' to 3'） | Reverse primer（5' to 3'） |
| --- | --- | --- |
| AtAHK2 | ATACGGACTCCGATGAATGG | CCATCTGCTTCGCATCAAG |
| AtAHK3 | GGTGTCAAGATTGGGCATCTC | CGTCGATCCCATTATCAACGA |
| AtCRE1 | ACATCACAAATTCGGAGTTCGA | GCTTGCTCTTAACGGTTTCATTATT |
| AtFT | CGCCAGAACTTCAACACTCG | TCTTCCTCCGCAGCCACT |
| AtActin2 | GGTAACATTGTGCTCAGTGGTGG | AACGACCTTAATCTTCATGCTGC |
| AtCRY1 | ATCTGGTTGTGGTTCTGGTGGTTG | TCAGGGTCATAAGGCATACTAAGA |
| AtCRY2 | GCTATCTGCTACAATCTCATCA | TCTTAGGGGAATCGGTTTA |
| AtFCA | CCCGTTAGGTGGTTATGGTGTTCC | TTGGTTTGGTTGCTGCATAGACTG |
| AtFD | CACCTCCTGCAACTGTTCTG | AGCCTCGAAAGAGGTGTTGA |
| AtFLC | CGGTCTCATCGAGAAAGCTC | CCACAAGCTTGCTATCCACA |
| AtFPA | CCACCAGCAGATAAGGCAAA | GTACCCTGACCATCCCCAGA |
| AtFVE | TCTCCTCAAGCAACGACACC | TGCGTTTTCTTCCCACTTTCT |
| AtGAI | GATTCGGCTTCTTCGTC | CGTCGCTGTAGTGGTTT |
| AtRGA | ACGGTGTTCGTTTAGTCCA | GCATCCGATTTGCTTCA |
| AtAP1 | ACAATATGCCTCCCCCTC | CTTCTTGATACAGACCACCC |
| AtCO | CTACAACGACAATGGTTCCATTAAC | CAGGGTCAGGTTGTTGC |
| AtLFY | AGATTATCTGTTCCACTTGTACGAACA | CGCCACGGTCTTTAGCAATT |
| AtSOC1 | TTCTCTTTCTTTCTTCTTCTCCCT | GTTAATTTCCCGATTGGCTAA |
| AtSPL3 | AGAGGCTTTGGAGAAGAAGCAGAAAGG | GAAGACCAGAGATCCGAACATGAGGAG |
| AtSPL9 | CAAGGTTCAGTTGGTGGAGGA | TGAAGAAGCTCGCCATGTATTG |
| AtSPY | GCTTCACAAGATTACACCCTC | AACTACCGCTGAATAAACCAC |
| AtTSF | CTCGGGAATTCATCGTATTG | CCCTCTGGCAGTTGAAGTAA |
| AtVRN1 | TGAAGATGAAGATGCCGAGGT | ACAAGGTTTGGATGGCTGAA |
| AtVRN3 | ATTGCCCTGCTGAATGATGG | ATGGCTGGTGGAATGTTGCT |
